# Root exudate metabolomes change under drought and show limited capacity for recovery

**DOI:** 10.1038/s41598-018-30150-0

**Published:** 2018-08-23

**Authors:** Albert Gargallo-Garriga, Catherine Preece, Jordi Sardans, Michal Oravec, Otmar Urban, Josep Peñuelas

**Affiliations:** 1 CSIC, Global Ecology Unit CREAF- CSIC-UAB, Bellaterra, 08193 Catalonia Spain; 2 CREAF, Cerdanyola del Vallès, 08193 Catalonia, Spain; 3 Global Change Research Institute, The Czech Academy of Sciences, Belidla 986/4a, CZ-60300 Brno, Czech Republic

## Abstract

Root exudates comprise a large variety of compounds released by plants into the rhizosphere, including low-molecular-weight primary metabolites (particularly saccharides, amino acids and organic acids) and secondary metabolites (phenolics, flavonoids and terpenoids). Changes in exudate composition could have impacts on the plant itself, on other plants, on soil properties (e.g. amount of soil organic matter), and on soil organisms. The effects of drought on the composition of root exudates, however, have been rarely studied. We used an ecometabolomics approach to identify the compounds in the exudates of *Quercus ilex* (holm oak) under an experimental drought gradient and subsequent recovery. Increasing drought stress strongly affected the composition of the exudate metabolome. Plant exudates under drought consisted mainly of secondary metabolites (71% of total metabolites) associated with plant responses to drought stress, whereas the metabolite composition under recovery shifted towards a dominance of primary metabolites (81% of total metabolites). These results strongly suggested that roots exude the most abundant root metabolites. The exudates were changed irreversibly by the lack of water under extreme drought conditions, and the plants could not recover.

## Introduction

The importance of rhizosphere processes is becoming increasingly recognised, and encompasses what happens in the zone of soil that is directly affected by roots and associated soil microorganisms. Such processes can include the acquisition of nutrients, growth inhibition of surrounding competitor plants (i.e. allelopathy), sending chemical signals to attract symbiotic partners (chemotaxis) such as between rhizobia and legumes, or in the promotion of the beneficial microbial colonization on root surfaces^1,2^. Several recent studies suggest a pivotal role of root exudates in rhizosphere function and thus in plant-microbe-soil relationships^3–5^. Root exudates comprise a large variety of compounds released by plants into the rhizosphere^1,6,7^, including low-molecular-weight primary metabolites (e.g. saccharides, amino acids, and organic acids), secondary metabolites (e.g. phenolics, flavonoids and terpenoids)^8,9^, and inorganic molecules (e.g. carbon dioxide and water)^3^. Root exudation is an important source of organic carbon in the soil and can account for up to 2–11% of total photosynthetic production^10–12^. Exudates can thus provide soil microorganisms, which are often carbon-limited, with an important energy source^3^. This leads to a process called “soil priming”, whereby the microbial community becomes more active and may liberate nutrients important for plants through its role in nutrient cycling^13^. Root exudates such as oxalic acid may also directly release organic compounds from organo-mineral aggregates, leading to greater microbial access to these compounds and increased net soil carbon loss^4^. Compounds in exudates also play important roles in various biological processes, including the mobilisation and acquisition of nutrients^14^, attracting and repelling certain microbial species^15–17^ and inhibiting the growth of competing plant species^18^.

The quantity and composition of root exudates is species-specific, can vary throughout the life of a plant^19,20^, and is affected by abiotic factors. For example, the availability of nutrients in the soil can affect exudation. Some plants in phosphorus (P) poor soils can exude higher amounts of organic acids and phosphatase that help to mobilise recalcitrant P, e.g. *Lupinus albus*^21,22^, *Medicago sativa*^23^, and *Brassica napus*^24^.

Increasing droughts are predicted for many regions worldwide^25,26^, including the Mediterranean Basin, the focus area of this study. Knowledge of the impacts of water stress on plants, soils and their interactions, including root exudation, is consequently vital. The majority of studies on the effects of drought on trees have focused on aboveground tissues and have neglected the impacts on belowground tissues^27–29^. This lack of information on roots, and root exudates in particular, is largely due to their inaccessibility, so methods tend to be labour-intensive and have low precision^29^. However, a large body of evidence now suggests that drought affects the growth and morphology of tree roots, including shorter lifespan, lower biomass, deeper rooting, and smaller diameter^29^. Root damage caused by drought tends to appear as reduced hydraulic conductivity and xylem embolism, eventually leading to root mortality under severe drought stress^30^.

The effects of drought stress on the quantity and quality of exudates, and the mechanisms of this phenomenon, are poorly understood, despite their potential importance. Previous work has found that the response of root exudation rate to drought is variable, depending on the intensity of the drought^31^, although a more recent experimental study found that increasing drought duration increased root exudation per unit root area in *Quercus ilex* (holm oak)^32^. Plants may up-regulate root exudation under moderate drought in order to release more mucilage, a polysaccharide that can ease the movement of roots through dry soil^33,34^. Exudate levels, however, appear to decrease under extreme drought, perhaps because plants redirect resources to essential processes. Inter- and intraspecific differences in exudation may account for differences in drought tolerance between species and individuals, and knowing how and why these differences occur may have practical applications for predicting and managing vegetation patterns in areas prone to drought.

Currently it is not well understood how much and how quickly root exudation recovers following a drought event. There are only two studies, to our knowledge, that have investigated this experimentally, with both indicating that the amount of exudation could return to pre-drought levels^32,35^. The recovery capacity of plant roots relating to root exudation has implications for the resilience of species, and even ecosystems, under increasing drought and requires more research.

Changes in exudate composition, as opposed to quantitative changes, may have impacts on the plant itself, on other plants, on soil properties (e.g. amount of soil organic matter), and on soil organisms, but very little is known about the effects of drought on exudate composition. It is known that the water-use efficiency of plants depends on nutrient availability in drought conditions^36–38^. P mobility decreases during drought stress and changes in exudate composition may help to mitigate this^39,40^. Drought-stressed plants can exude more organic acids^40,41^, which have a role in the mobilisation of P, thus improving the potential for nutrient release from the soil. Therefore, it is important to gain knowledge on the possible change in root exudate composition in Mediterranean plants under increasing drought, due to the role of exudates in plant nutrient uptake capacity.

Metabolomic techniques have recently been used to investigate the composition of exudates in great detail^42^. Important compounds identified in root exudates include organic acids from primary and secondary metabolism (such as oxalate, malate, salicylate, ascorbate, lactate, and citrate), saccharides and their derivatives, and amino acids^43,44^. Analyses of the metabolic profiles of root exudates have demonstrated great plasticity in metabolite composition linked with abiotic (P and iron soil deficiency) or biotic (inter-specific competition) shifts^43,44^. One study found changes in the exudate composition of soybean (*Glycine max*) under drought stress, including an increase in osmolytes (including proline and pinitol)^45^, which can assist in the maintenance of cell turgor. Interestingly, in that study there were positive correlations between the composition of exudates and phloem (in relation to organic acids and amino acids) and between exudates and roots (in relation to saccharides)^45^. This suggests that drought effects on the metabolic profile of the phloem and roots, which may serve to achieve osmotic adjustment within the plant, can later change the metabolic profile of root exudates. To the best of our knowledge, however, metabolomic analyses have not been used to study root exudates under multiple levels of water availability and especially under drought stress and the subsequent recovery.

This study focused on the identification of compounds^46–50^ in the root exudates of holm oak (*Quercus ilex*) under varying drought conditions and on their role in the interactions between plants and soil. *Q. ilex* is a widespread Mediterranean tree species that is generally drought tolerant but has shown measurable drought responses to severe water stress in previous field experiments^51–55^, although usually followed by recovery, indicating a capacity to adapt to this stress. Individuals of *Q. ilex* were grown in a greenhouse experiment under drought conditions of different intensities, and the metabolites in the semipolar fraction of exudates were identified by untargeted profiling using liquid chromatography–mass spectrometry (LC–MS). Exudate composition was also analysed following a subsequent recovery period. Our overall objective was to assess the changes in composition of exudates due to drought stress and the corresponding recovery. We proposed three hypotheses. (1) In the exudates of drought-treated plants there would be an increase in the proportion of compounds associated with drought stress, such as the plant hormone abscisic acid and other secondary metabolites, and that the changes in composition would increase as drought intensity increased. (2) The composition of exudates following a recovery period will be similar to the pre-drought composition. (3) The level of recovery will depend on the level of drought, with exudate composition in plants after recovery from low to moderate drought returning to a composition similar to that of the controls. However, the exudate composition of plants recovering from severe levels of drought may not be so similar to the controls due to severe root damage.

## Results

### Effect of drought on chlorophyll fluorescence of leaves

**Figure 1:**
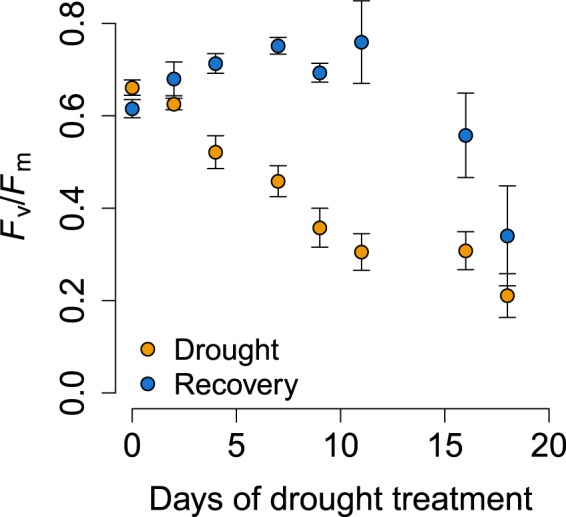
Changes in mean maximum photochemical efficiency of photosystem II (*F*_v_/*F*_m_) for *Quercus ilex* in response to (in yellow) the drought treatment (8 levels) and (in blue) the subsequent 6 week recovery period. Nine plants measured for each drought level. Error bars are one SE.

Values of the maximum photochemical efficiency of photosystem II, *F*_v_/*F*_m_, (where *F*_m_ is maximum fluorescence, and *F*_v_ is the difference between *F*_m_ and the minimum fluorescence, *F*_o_) decreased through the drought experiment, demonstrating a decrease in the photosynthetic quantum efficiency of photosystem II, and therefore higher stress (Fig. 1). Values recovered in the re-watering period except for in the two highest drought treatments (Fig. 1).

### Effect of soil treatment on exudate composition

The metabolomic composition of exudates collected from plants grown under the different soil treatments (control, drought-stressed and sterilised) was not significantly different (*P* > 0.1), therefore for all subsequent analyses the samples from different soil treatments were pooled together.

### Effect of drought on exudate composition

**Figure 2:**
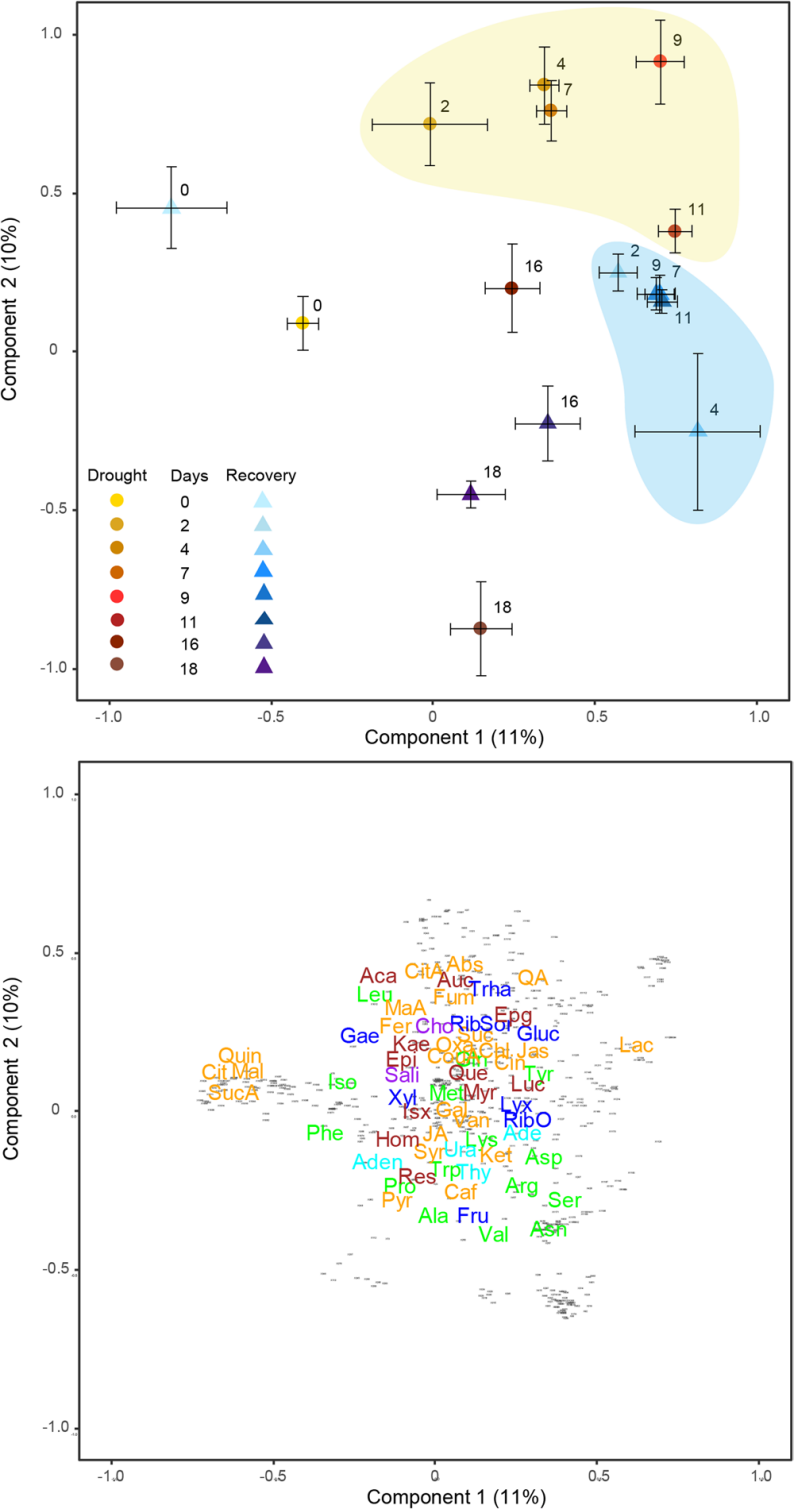
Plots of cases and variables in the PLSDA conducted with the metabolomic variables of *Quercus ilex* using Component 1 versus Component 2. (**a**) Scores categorised by each experiment * drought level (mean ± SE). Drought duration increases from zero to 18 days. The yellow shading is grouping the drought treatment and the blue shading is grouping the recovery treatment. (**b**) Loadings of the metabolomic variables in Components 1 and 2. The various metabolomic families are represented by colours: dark blue, saccharides; green, amino acids; orange, compounds involved in the metabolism of amino acids and saccharides; cyan, nucleotides; brown, phenolics; and red, others. Metabolites: arginine (Arg), asparagine (Asn), aspartic acid (Asp), glutamic acid (Glu), glutamine (Gln), isoleucine (Ile), lysine (Lys), leucine (Leu), methionine (Met), phenylalanine (Phe), serine (Ser), tryptophan (Trp), threonine (Thr), tyrosine (Tyr), valine (Val), adenine (Ade), adenosine (Aden), uracil (Ura) thymidine (Thy), chlorogenic acid (CGA), trans-caffeic acid (Caf), α-ketoglutaric acid (KG), citric acid (Cit), L-malic acid (Mal), lactic acid (Lac), abscisic acid (Abs) pyruvate (Pyr), succinic acid (SAD), pantothenic acid hemicalcium salt (Pan), jasmonic acid (JA), 5,7-dihydroxy-3,4,5–trimethoxyflavone (Fla), acacetin (AC), epicatechin (EC), epigallocatechin (EGC), homoorientin (Hom), isovitexin (Ivx), kaempferol (Kae), myricetin (Myr), quercetin (Qct), resveratrol (Rvt), saponarin (Sp), catechin hydrate (Cat), 3-coumaric acid (CouA), gallic acid (GA), quinic acid (QuiA), sodium salicylate (Sal), syringic acid (Syr), trans-ferulic acid (Fer), vanillic acid (Van), 2-deoxy-D-ribose (RibO), D-(−)-lyxose (Lyx), galactose (Gae), glucose (Glu), fructose (Fru), xylose(Xyl), D-(+)-sorbose (Sor), D-(+)-trehalose dehydrate (Trha), aucubin (Auc).

**Figure 3:**
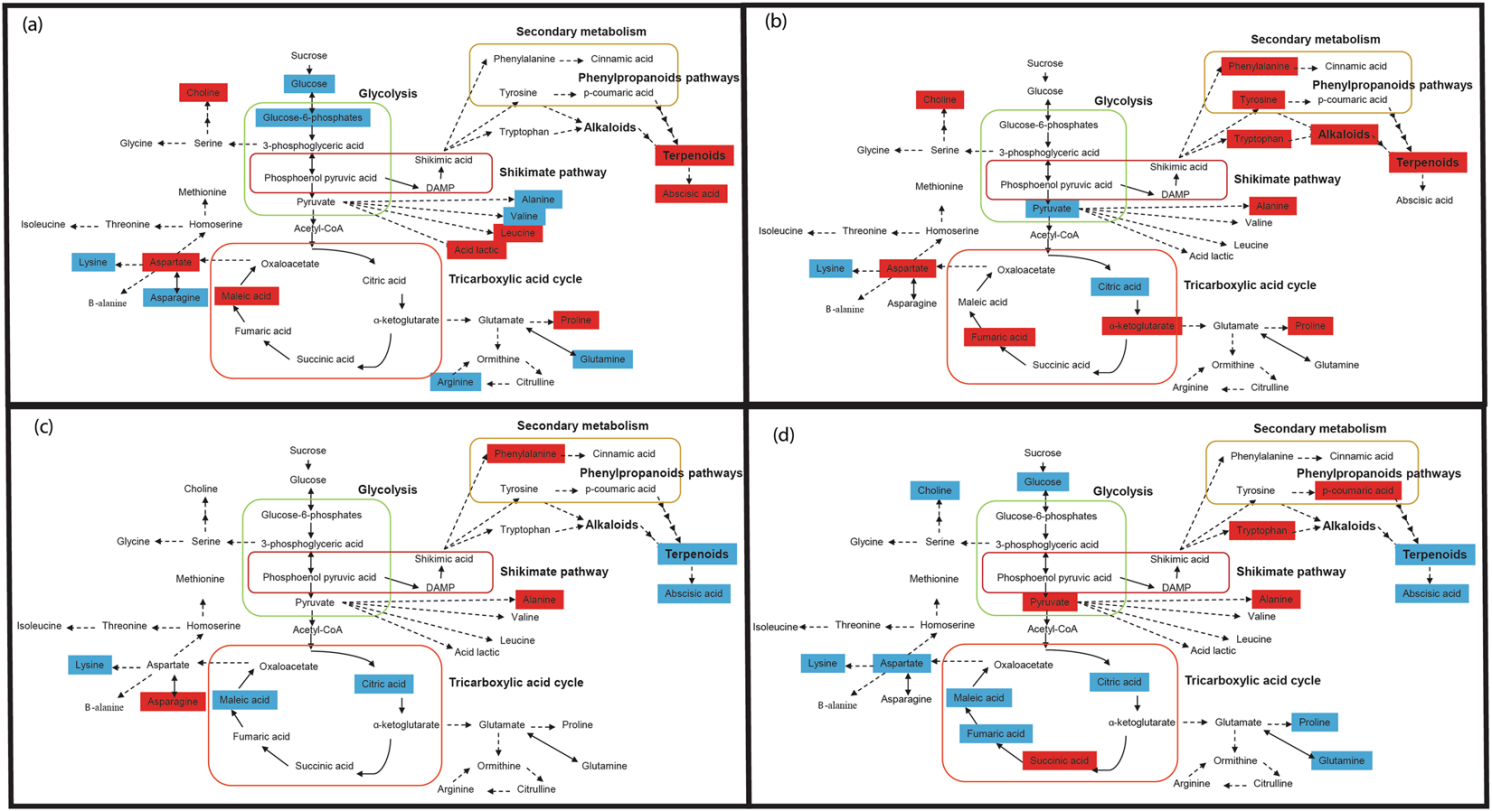
Response of individual metabolites in representative metabolic pathways to drought treatments measured as the fold difference between individual treatments and that in the recovery treatment. Red colour indicates activated (with increased concentrations) metabolites and blue colour indicates deactivated (with decreased concentrations) metabolites. (**a**) Metabolites of different metabolic pathways that shifted their concentrations under the mean drought (2, 4, 7, 9, 11 days with no water). (**b**) Metabolites of different metabolic pathways that shifted their concentrations under the most extreme drought (18 days with no water). (**c**) Metabolites of different metabolic pathways that shifted their concentrations from the mean of drought (2, 4, 7, 9, 11 days with no water) to the mean of the recovery (2, 4, 7, 9, 11 days with water). (**d**) Metabolites of different metabolic pathways that shifted their concentrations from the most extreme drought (18 days with no water) to recovery.

We detected 601 metabolites, 63 of which were identified. Amino acids were the most abundant compounds, followed by carbohydrates and organic acids. The metabolomes of the two phases of the control treatment (Drought 0 and Recovery 0 in Fig. 2a) were very similar (*P* > 0.1 in both axes), as expected. Drought intensity had a strong effect on exudate composition (*P* < 0.001). Drought-stressed plants (except for those with the longest drought) were displaced along Component 1 relative to the control (Drought 0 and Recovery 0) towards higher concentrations of lactic acid, pyruvate, abscisic acid (ABA), and glucose (Fig. 2). Overall, exudates under drought consisted mainly of secondary metabolites (71% of total metabolites). The metabolomic composition under the most extreme drought (18 d with no water) and the high extreme (16 d with no water) changed mostly along Component 2, towards higher concentrations of fructose, arginine, and valine. The one-way ANOVA (Table 1 and Fig. 3) indicates higher concentrations of the compounds related with the synthesis of alkaloids and terpenoids such as the precursors, phenylalanine, tyrosine and tryptophan for these two drought levels. In the case of the two most extreme drought treatments (16 and 18 days), lower concentrations, indicating down-regulation, were measured for adenosine, lysine and jasmonic acid, with pyruvate and citric acid also showing lower concentrations in the most extreme treatment (18 days) (Table 2 and Fig. 3).

**Table 1:** Compounds that were activated and deactivated in the drought and recovery plants for the 2–16 d droughts, as compared with the control (0 days of drought).

Drought		Recovery	
Deactivated	Activated	Deactivated	Activated
Valine (7%, *P* < 0.01)	Abscisic acid (50%, *P* < 0.01)	Abscisic acid (32%, *P* < 0.01)	Valine (9%, *P* < 0.01)
Arginine (28%, *P* < 0.01)	Leucine (14%, *P* < 0.01)	Leucine (14%, *P* < 0.001)	Arginine (32%, *P* < 0.001)
Fructose (29%, *P* < 0.001)	Acacetin (15%, *P* < 0.001)	Acacetin (19%, *P* < 0.01)	Fructose (31%, *P* < 0.01)
Ribose (12%, *P* < 0.01)	Malic acid (13%, *P* < 0.01)	Proline (13%, *P* < 0.01)	Ribose (19%, *P* < 0.01)
Alanine (13%, *P* < 0.001)	Proline (20%, *P* < 0.001)	Choline (35%, *P* < 0.01)	Alanine (14%,*P* < 0.001)
Lysine (24%, *P* < 0.01)	Choline (47%, *P* < 0.01)		Adenine (23%, *P* < 0.001)
Asparagine (16%, *P* < 0.01)	Homoorientin (19%, *P* < 0.001)		
Uracil (21%, *P* < 0.01)			
Glutamine (17%, *P* < 0.001)			
Methionine (12%, *P* < 0.01)			

The plants from the longest drought (18 d) did not recover and thus have not been included in this table. This table shows only the metabolites activated and deactivated in all the treatments of drought (and corresponding recoveries) between 2 to 16 days. In brackets % of each compound change in front of the control and the *P* values.

**Table 2:** Metabolites (**a**) deactivated and (**b**) activated in each specific level of drought (in unwatered plants) compared with the control.

Drought 2	Drought 4	Drought 7	Drought 9	Drought 11	Drought 16	Drought 18
**(a) Deactivated**						
Methylthio-ribose (13%, *P* < 0.01)	Valine (35%, *P* < 0.01)	Myricetin (9%, *P* < 0.01)	Lucenin (11%, *P* < 0.01)	Ketoglutaric Acid (66%, *P* < 0.01)	Adenosine (9%, *P* < 0.01)	Adenosine (18%, *P* < 0.01)
Alanine (6%, *P* < 0.01)	Cinnamic acid (30%, *P* < 0.01)	Coumaric acid (10%, *P* < 0.01)	Glutamine (5%, *P* < 0.01)	Isoleucine (7%, *P* < 0.01)	Lysine (26%, *P* < 0.01)	Lysine (8%, *P* < 0.01)
Arginine (15%, *P* < 0.01)	Ribose (8%, *P* < 0.01)	Fructose (31%, *P* < 0.001)	Xylose (13%, *P* < 0.01)	Succinic acid (8%, *P* < 0.01)	Jasmonic acid (30%, *P* < 0.001)	Jasmonic acid (48%, *P* < 0.001)
Sorbose (19%, *P* < 0.01)	Homoorientin (8%, *P* < 0.01)	Cinnamic acid (15%, *P* < 0.01)	Ribose (21%, *P* < 0.01)	Coumaric acid (10%, *P* < 0.01)		Pyruvate (12%, *P* < 0.01)
Jasmonic acid (27%, *P* < 0.001)	Glucose (13%, *P* < 0.01)	Ketoglutaric acid (30%, *P* < 0.01)	Asparagine (7%, *P* < 0.05)	Jasmonic acid (46%, *P* < 0.001)		Citric acid (23%, *P* < 0.01)
Valine (21%, *P* < *P* < 0.01)	Tyrosine (17%, *P* < 0.01)	Tryptophan (15%, *P* < 0.01)	Vanillic acid (21%, *P* < 0.01)	Ribose (12%, *P* < 0.01)		
Tryptophan (29%, *P* < 0.001)	Isoleucine (6%, *P* < 0.01)	Acacetin (12%, *P* < 0.01)	Arginine (27%, *P* < 0.01)	Tyrosine (8%, *P* < 0.01)		
Lyxose (25%, *P* < 0.01)	Myricetin (11%, *P* < *P* < 0.01)	Quercetin (34%, *P* < 0.01)	Epicatechin (21%, *P* < 0.001)	Malic acid (18%, *P* < 0.01)		
Fructose (8%, *P* < 0.01)	Fructose (21%, *P* < 0.01)	Alanine (11%, *P* < 0.01)	Isovitexin (9%, *P* < 0.01)	Thymidine (31%, *P* < 0.01)		
Myricetin (10%, *P* < 0.01)	Lysine (12%, *P* < 0.01)	Isoleucine (7%, *P* < 0.01)	Uracil (31%, *P* < 0.01)	Alanine (12%, *P* < 0.01)		
	Galactose (15%, *P* < 0.01)	Ribose (11%, *P* < 0.01)	Kaempferol (31%, *P* < 0.01)			
		Epigallocatechin (7%, *P* < 0.01)	Oxaloglutarate (42%, *P* < 0.01)			
		Gallic acid (55%, *P* < 0.01)	Methionine (9%, *P* < 0.01)			
			Chlorogenic acid (18%, *P* < 0.01)			
			Galactose (35%, *P* < 0.001)			
			Lactic acid (17%, *P* < 0.01)			
			Lyxose (30%, *P* < 0.001)			
**(b) Activated**						
Acacetin (12%, *P* < 0.01)	Abscisic acid (40%, *P* < 0.001)	Abscisic acid (65%, *P* < 0.001)	Abscisic acid (37%, *P* < 0.01)	Lyxose (7%, *P* < 0.01)	Choline (38%, *P* < 0.01)	Homoorientin (31%, *P* < *P* < 0.01)
Leucine (43%, *P* < *P* < 0.01)	Malic acid (37%, *P* < 0.001)	Glucose (17%, *P* < 0.01)	Acacetin (17%, *P* < 0.01)	Leucine (23%, *P* < 0.01)	Proline (28%, *P* < 0.01)	Fumarate (7%, *P* < 0.01)
Homoorientin (24%, *P* < 0.01)	Leucine (10%, *P* < 0.01)	Malic acid (44%, *P* < 0.01)	Malic acid (26%, *P* < 0.01)	Vanillic acid (43%, *P* < 0.01)	Tyrosine (22%, *P* < 0.01)	Acacetin (11%, *P* < 0.01)
Abscisic acid (28%, *P* < 0.01)	Acacetin (9%, *P* < 0.01)	Proline (20%, *P* < 0.01)	Leucine (25%, *P* < 0.01)	Valine (19%, *P* < 0.01)		Tryptophan (33%, *P* < 0.01)
Malic acid (56%, *P* < 0.01)	Coumaric acid (8%, *P* < 0.01)	Sorbose (15%, *P* < 0.01)	Adenine (9%, *P* < 0.01)	Pyruvate (18%, *P* < 0.01)	Abscisic acid (48%, *P* < 0.01)	Epigallocatechin (26%, *P* < 0.01)
Proline (18%, *P* < 0.01)	Proline (7%, *P* < 0.01)	Quinic acid (11%, *P* < 0.01)	Fructose (11%, *P* < 0.01)	Acacetin (11%, *P* < 0.01)	Tryptophan (6%, *P* < 0.01)	Alanine (41%, *P* < 0.01)
Isoleucine (6%, *P* < 0.01)	Sorbose (50%, *P* < 0.01)	Leucine (23%, *P* < 0.01)	Ketoglutaric acid (18%, *P* < 0.01)	Arginine (21%, *P* < 0.03)	Epigallocatechin (9%, *P* < 0.01)	Vanillic acid (30%, *P* < 0.01)
Galactose (18%, *P* < 0.01)	Citric acid (16%, *P* < 0.01)	Citric acid (19%, *P* < 0.01)	Tryptophan (25%, *P* < 0.01)	Galactose (19%, *P* < 0.01)	Homoorientin (16%, *P* < 0.01)	Aspartate (21%, *P* < 0.01)
Lactic acid (36%, *P* < 0.01)	Aspartate (32%, *P* < 0.01)		Methylthioribose (9%, *P* < 0.01)	Chlorogenic acid		Choline (42%, *P* < 0.01)
Chlorogenic acid (15%, *P* < 0.01)	Adenine (24%, *P* < 0.01)		Myricetin (6%, *P* < 0.01)	Homoorientin (24%, *P* < 0.01)		Proline (20%, *P* < 0.01)
Gallic acid (58%, *P* < 0.01)	Aucubin (20%, *P* < 0.01)		Adenosine (6%, *P* < 0.01)	Gallic acid (65%, *P* < 0.01))		Tyrosine (6%, *P* < 0.01)
Adenine (25%, *P* < 0.01)						Ketoglutaric acid (55%, *P* < 0.01)
Trehalose dihydrate (19%, *P* < 0.01)						Phenylalanine (24%, *P* < 0.01)
Oxaloglutarate (17%, *P* < 0.01)						
Xylose (21%, *P* < 0.01)						

Each level of drought was indicated by the number of days without water.

Some metabolites, particularly ABA, aspartic acid and leucine (amino acids), and acacetin (a flavonoid compound) were found in higher concentrations in the root exudates of the plants under drought stress and their concentrations decreased thereafter during the recovery (Table 1). Some compounds such as leucine and acacetin were found at highest concentrations at lower levels of drought, but not at the most extreme drought stress (Fig. 3). The synthesis of the terpenoids was increased during drought, however the glycolysis pathway was down-regulated (Fig. 3).

### Effect of recovery on exudate composition

The metabolomes collected after recovery from the various drought durations (except the plants with the longest drought duration of 18 days) shifted towards higher concentrations of several amino acids (aspartate, asparagine, arginine, and valine) and pyruvate but not towards the same metabolomic profile of the controls (Fig. 2). Overall, the metabolite composition under recovery was dominated by primary metabolites (81% of total metabolites). The composition of the exudates thus clearly differed between the drought and recovery pots (*P* < 0.01), even when plants had experienced low levels of drought. The exudates of the recovery plants were characterised by higher valine and arginine concentrations than the exudates of drought (Tables 2 and 3). This recovery response in exudate composition, with clear differences from pre-drought metabolomes, was in contrast to the recovery of the leaves, shown by *F*_v_/*F*_m_ measurements, which returned to pre-drought levels except for in the two highest drought treatments (Fig. 1

**Table 3:** Metabolites (**a**) Deactivated and (**b**) activated in each specific level of recovery from the corresponding level of drought (in re-watered plants) compared with the control.

Recovery 2	Recovery 4	Recovery 7	Recovery 9	Recovery 11	Recovery 16	Recovery 18
(a) Deactivated						
Aspartate (27%, *P* < 0.01	Resveratrol (11%, *P* < 0.01)	Homoorientin (11%, *P* < 0.01)	Quercetin (27%, *P* < 0.01)	Abscisic acid (65%, *P* < 0.01)	Abscisic acid (45%, *P* < 0.01)	Acacetin (24%, *P* < 0.01)
Succinic acid (65%, *P* < 0.0001)	Coumaric acid (5%, *P* < 0.01)	Malic acid (18%, *P* < 0.01)	Abscisic acid (37%, *P* < 0.01)	Isoleucine (15%, *P* < 0.01)	Glucose (42%, *P* < 0.01)	Adenine (12%, *P* < 0.01)
Sodium salicylate	Adenosine (17%, *P* < 0.01)	Choline (36%, *P* < 0.01)	Gallic acid (12%, *P* < 0.01)	Tyrosine (11%, *P* < 0.01)	Malic acid (39%, *P* < 0.01)	Malic acid (27%, *P* < 0.01)
Gallic acid (16%, *P* < 0.01)	Homoorientin (8%, *P* < 0.01)	Tyrosine (7%, *P* < 0.01)	Proline (6%, *P* < 0.01)	Quercetin (29%, *P* < 0.01)	Quinic acid (21%, *P* < 0.01)	Abscisic acid (52%, *P* < 0.01)
	Isovitexin (7%, *P* < 0.01)	Leucine (32%, *P* < 0.01)			Adenine (13%, *P* < 0.01)	Gallic acid (19%, *P* < 0.01)
	Glutamine (6%, *P* < 0.01)	Abscisic acid (21%, *P* < 0.01)			Trehalose dehydrate (32%, *P* < 0.01)	Lysine (6%, *P* < 0.01)
	Sorbose (8%, *P* < 0.01)	Phenylalanine (15%, *P* < 0.01)			Aucubin (15%, *P* < 0.01)	Ferulic acid (23%, *P* < 0.01)
	Lyxose (13%, *P* < 0.01)				Thymidine (11%, *P* < 0.01)	Citric acid (31%, *P* < 0.01)
	Asparagine (20%, *P* < 0.01)				Proline (12%, *P* < 0.01)	Epigallocatechin (7%, *P* < 0.01)
	Xylose (28%, *P* < 0.01)				Acacetin (23%, *P* < 0.01)	Chlorogenic acid (32%, *P* < 0.01)
	Jasmonic acid (34%, *P* < 0.01)				Chlorogenic acid (21%, *P* < 0.01)	
	Trehalose dehydrate (28%, *P* < 0.01)				Choline (35%, *P* < 0.01)	
	Aspartic acid (20%, *P* < 0.01)				Fumarate (8%, *P* < 0.01)	
	Tyrosine (11%, *P* < 0.01)				Lyxose (11%, *P* < 0.01)	
	Abscisic acid (33%, *P* < 0.01)				Vanillic acid (13%, *P* < 0.01)	
	Succinic acid (55%, *P* < 0.0001)				Ferulic acid (12%, *P* < 0.01)	
	Isoleucine (17%, *P* < 0.01)				Glutamine (9%, *P* < 0.01)	
	Uracil (24%, *P* < 0.01)					
**(b) Activated**						
Quercetin (30%, *P* < 0.01)	Quercetin (28%, *P* < 0.01)	Valine (21%, *P* < *P* < 0.01)	Malic acid (34%, *P* < 0.01)	Pyruvate (14%, *P* < 0.01)	Homoorientin (11%, *P* < 0.01)	Alanine (24%, *P* < 0.01)
Arginine (28%, *P* < 0.01)	Arginine (44%, *P* < 0.01)	Gallic acid (34%, *P* < 0.01)	Lyxose (9%, *P* < 0.01)	Coumaric acid (23%, *P* < 0.01)	Hydroxyphenyl-pyruvate	Myricetin (8%, *P* < 0.01)
Cinnamic acid (21%, *P* < 0.01)	Ferulic acid (21%, *P* < 0.01)	Lysine (15%, *P* < 0.01)	Tyrosine (6%, *P* < 0.05)	Vanillic acid (29%, *P* < 0.01)	Fructose (23%, *P* < 0.01)	Quercetin (18%, *P* < 0.01)
Ferulic acid (7%, *P* < 0.01)	Ribose (19%, *P* < 0.01)	Lucenin (8%, *P* < 0.01)	Lysine (26%, *P* < 0.01)	Myricetin (8%, *P* < 0.01)	Myricetin (10%, *P* < 0.01)	Homoorientin (18%, *P* < 0.01)
Pyruvate (34%, *P* < 0.01)		Aspartic acid (12%, *P* < 0.01)	Valine (49%, *P* < 0.01)	Gallic acid (11%, *P* < 0.01)	Adenosine (18%, *P* < 0.01)	Asparagine (12%, *P* < 0.01)
		Glucose (18%, *P* < 0.01)	Vanillic acid (17%, *P* < 0.01)	Alanine (27%, *P* < 0.01)	Tryptophan (14%, *P* < 0.01)	Caffeic acid (32%, *P* < 0.01)
		Oxaloglutarate (59%, *P* < 0.01)	Thymidine (16%, *P* < 0.01)	Valine (21%, *P* < 0.01)	Succinic acid (34%, *P* < 0.01)	Phenylalanine (10%, *P* < 0.01)
		Quinic acid (22%, *P* < 0.01)	Epigallocatechin (33%, *P* < 0.01)	Tryptophan (8%, *P* < 0.01)	Coumaric acid (13%, *P* < 0.01)	Adenosine (14%, *P* < 0.01)
		Arginine (28%, *P* < 0.01)	Pyruvate (42%, *P* < 0.01)	Lyxose (9%, *P* < 0.01)		Methionine (11%, *P* < 0.01)
		Citric acid (23%, *P* < 0.01)	Ketoglutaric acid (22%, *P* < 0.01)	Epigallocatechin (32%, *P* < 0.01)		Isovitexin (9%, *P* < 0.01)
		Methionine (16%, *P* < 0.01)	Myricetin (8%, *P* < 0.01)	Asparagine (31%, *P* < 0.01)		
		Chlorogenic acid (21%, *P* < 0.01)	Methionine (10%, *P* < 0.01)	Fructose(32%, *P* < 0.01)		
		Ribose (21%, *P* < 0.01)	Aucubin (9%, *P* < 0.01)	Oxaloglutarate (11%, *P* < 0.01)		
		Glutamine (8%, *P* < 0.01)	Glucose (36%, *P* < 0.01)	Ketoglutaric acid (8%, *P* < 0.01)		
		Lyxose (44%, *P* < 0.01)	Ribose (22%, *P* < 0.01)	Ribose (14%, *P* < 0.01)		
		Resveratrol (15%, *P* < 0.01)	Asparagine (55%, *P* < 0.01)			
		Sorbose (14%, *P* < 0.01)	Cinnamic acid (19%, *P* < 0.01)			
			Kaempferol (7%, *P* < 0.01)			

Each level of recovery was indicated by the previous number of days without water.

).

The interaction between drought level and drought/recovery was significant (*P* < 0.05), indicating that the changes in exudate composition associated with recovery depended on the intensity of the earlier drought. Specifically, exudate metabolomes for the recovery plants generally clustered together, except those for the longest drought (Recovery 18) (Fig. 2a), which were slightly separated from the other recovery plants, displaced along Component 1 in the direction of the controls and the drought-stressed plants at the same drought level (Drought 18).

## Discussion

The effect of drought on the total metabolites contained within root exudates has not yet been extensively investigated, but, could be important in a context of increasing aridity in several world areas such as the Mediterranean Basin^25,26^. This metabolomic analysis of root exudates during drought and recovery indicated that drought stress has a strong effect on the composition of *Q. ilex* exudates. Most of the metabolites that increased in concentration under drought and decreased in concentration under recovery (Table 1) were consistent with those previously reported^56,57^. Plant exudates under all drought levels except the highest (18 d) shifted towards a higher expression of secondary compounds (including antioxidants) and soluble sugars, plus compounds that act as osmolytes, thus compounds associated with the response of plants to drought stress. The metabolite composition of the root exudates under recovery shifted towards a composition dominated mainly by some amino acids and with decreases in the majority of saccharides and secondary compounds.

### Effect of drought on exudate composition

The compounds that were most activated (those with the highest concentration) by the drought treatment were abscisic acid (ABA), choline, terpenoids, proline, aspartate, leucine, acacetin and maleic acid. The composition of root exudates has rarely been studied; a recent review indicated that only nine studies have reported metabolomic analyses of exudates^42^. The compounds reported in these studies predominantly included primary metabolites such as saccharides, organic acids, and amino acids but also various secondary metabolites such as phenolic compounds and phytohormones^42^ similar to our study.

The increase in concentration of ABA was one of the most significant increases during the drought treatment, compatible with its characterisation as a universal plant stress hormone. An increase in the concentration of ABA in the roots is a very consistent physiological change in plants under drought^37,58,59^, although the predominant site of synthesis within the plant (roots or leaves) is not clear^60–62^. ABA reduces growth under drought conditions^29,59^ and leads to stomatal closure^58,60^. ABA in roots also mediates an enhancement of the biosynthesis of osmolytes, such as the amino acid proline, protective proteins^63,64^, and organic acids such as malic, lactic, aspartic, and fumaric acids that are also associated with osmolytic function and thus water retention^65^. Our results strongly suggest that root exudate metabolite composition reflects the composition of the rest of the plant, with plants producing exudates using the most abundant metabolites under the current environmental conditions^6,45,66,67^. Therefore, as high levels of ABA within roots are predicted during drought, then high levels of ABA in the exudates should also be expected. Relating to other organic acids, the concentration of maleic acid is also increased under drought, as also reported in root exudates of maize plants under drought conditions^40^.

The concentrations of the amino acids aspartate and leucine were the second and third most increased concentrations in the drought-stressed plants. A typical effect of drought is the accumulation of solutes, such as amino acids and saccharides, within plant leaves and roots, which has been reported in many studies^68–70^. This accumulation, sometimes termed osmotic adjustment, may help to maintain cell turgor and reduce water loss from cells^68^. A net benefit to the plant that increases fitness in water-stressed environments has not been firmly established^71,72^, but evidence suggests that solute accumulation may help to maintain root development to access deeper reserves of water^72^. A recent review also reported a positive correlation between osmotic accumulation and plant productivity^73^. Previous studies of the effect of drought stress on the foliar metabolome of various species, including *Q. ilex* (the species used in our experiment)^53^, *Triticum aestivum* (wheat)^74^, and two common grass species^37,38^, have found changes in foliar metabolite composition indicating enhanced osmoprotection. Also, a recent paper investigating the effects of drought on the metabolome of barley (*Hordeum vulgare*) leaves and roots reported increases in osmoprotectants and antioxidants^75^. Our finding of increased concentrations of these amino acids indicates that the accumulation of solutes that has been shown in leaves and roots also occurs in root exudates, perhaps indicating the general osmotic status of the roots.

At the most severe levels of drought in our study, there was an increase in the exudation of proline, adenine, cinnamic acid, and homoorientin. Proline synthesis has been up-regulated in several plant species under drought^76^ and other circumstances such as after wounding^64^, acting by buffering the cellular redox status during drought. Adenine is a precursor of several cytokinins, secondary compounds associated with triggering several anti-drought mechanisms in plants^77,78^. The flavonoids acacetin and homoorientin (O-methylated flavones) were also present in higher concentrations during drought. Flavonoids are secondary metabolites with an antioxidant function in terrestrial plants and accumulate during stressful environmental conditions^79–81^. Flavonoids play a direct role in drought tolerance^82^ and are associated with symbiosis, signalling, plant development, and plant defences^83–86^, and the differential accumulation of specialised metabolites in border cells likely serves important defensive and signalling roles^87^. Vanillic acid is an organic acid that was present in higher concentrations in the drought samples in our study, as also reported in exudates of drought-stressed crested wheatgrass (*Agropyron cristatum*)^41^.

Overall, our results strongly suggest that plants produce exudates using the most abundant metabolites under the current environmental conditions. This agrees with a previous study that found strong links between exudate composition and the composition of both phloem (relating to organic acids and amino acids) and root biomass (relating to saccharides)^45^. Also supporting our theory is the observation that exudation is generally believed to be a mostly passive process^6^ and a recent study found that there was a weak positive correlation between the amount of some saccharides (fructose, glucose and sucrose) exuded and the concentration in the root tissue. This suggests that these compounds are released by passive or facilitated diffusion and that the amount of these compounds present in exudates reflects that of the roots^66^. In contrast, there is recent evidence showing that organic acids and amino acids appear to be mostly actively exuded from roots^66,67^ meaning that the metabolite composition of these compounds might not necessarily be expected to reflect that of the rest of the plant.

It is also important to note that there can be differences in the drought response of metabolites in different organs of the plant, for example one study with the highly drought tolerant plant *Caragana korshinskii* showed that the metabolites that were found to increase under drought varied between the leaves, stem, root collar and roots, reflecting the higher drought tolerance of the stem and roots compared with the leaves^88^. Similarly, a study with two common grass species (*Holcus lanatus* and *Alopecurus pratensis*) revealed opposite metabolic responses to drought between shoots and roots. While roots remained active, with a high amount of primary metabolites, leaves showed a decrease in primary metabolism and an increase in some secondary metabolites (such as organic acids, terpenes and phenols)^37,38^. This response of the shoot seems to reflect more the drought response we saw in root exudates. Therefore, there is potentially a complicated interplay between the effect of the leaves and the roots on the composition of exudates, with the leaves having a greater impact during drought and root metabolism being more important during recovery and non-drought times.

### Effect of recovery on exudate composition

The longest drought had an irreversible effect on the metabolome as after six weeks of re-watering the exudates did not return to the pre-drought composition. This is in contrast to recent work in the same study system that showed that the amount of carbon exuded following drought was able to recover, even after extreme drought stress^32^. The compounds that showed the largest increase in concentration during recovery from drought included several amino acids such as alanine, arginine, valine and asparagine, one intermediate of saccharides and amino acid metabolism, which was citrate, and two saccharides, fructose and ribose. This is partially consistent with a recovery of energy production capacity and protein synthesis and thus growth. Saccharides and amino acids are typical semi-polar primary metabolites that should be common in growing plant tissues, such as fine roots of young tree saplings, under conditions of no stress. The main precursor metabolite in glycolysis is a fructose. The yield of glycolysis is ATP and NADH with pyruvate as an end product. Citrate is an important intermediary in the tricarboxylic acid cycle and also serves as a substrate for the cytosolic production of acetyl-CoA. CoA is important in numerous metabolic processes, especially in providing carbon substrates for secondary metabolism. The levels of saccharides, pyruvate and citrate (precursors for CoA biosynthesis), including amino acids alanine, isoleucine and valine were higher in the recovery samples (Table 3). Overall, the dominance of compounds involved in primary metabolism, typical of a growing plant, may again suggest that exudates mainly contain the most abundant metabolites in the fine roots at each moment, depending on the current plant metabolic status. Phenylpropanoids are the precursors of flavonoids, isoflavonoids, anthocyanins, and lignin and are synthesised from the primary amino acid phenylalanine. The conversion of phenylalanine to cinnamic acid by phenylalanine ammonia-lyase is an early step in the biosynthesis of these compounds. Phenylalanine was abundant in both the drought and recovery samples but was higher in the recovery samples (Tables 2 and 3).

Concentrations of many amino acids were much higher in the recovery than the drought samples (Table 3). Asparagine was the most abundant amino acid in the recovery samples and increased relative to the drought samples. Asparagine is an end-point amino acid that serves as a major compound for transporting and storing nitrogen in plant cells^89^.

Even for plants exposed to only very mild levels of drought, exudation still differed in composition compared to those in the control drought treatment after six weeks of re-watering, demonstrating that even short-term droughts can lead to long-term measurable changes. This revealed a clear difference with the above-ground response as the observed leaf chlorophyll fluorescence, values returned to normal, pre-drought, levels for plants at all drought treatments except for the two most severe ones. Chlorophyll fluorescence has been shown to be sensitive to photosynthetic drought and recovery in the study species^90^, however other studies have revealed that photosynthesis and chlorophyll fluorescence measurements are not always coupled^91,92^. Therefore, it is possible that the aboveground part of the plant was still affected by the drought, despite this not being shown by our measurements of chlorophyll fluorescence. This highlights the importance of investigating the below-ground processes under drought, and root exudates in particular, as responses may be in contrast to what is seen above-ground.

At the highest drought intensity there was no evidence of any recovery of the exudate metabolome, and the re-watered plants had exudate metabolomes that closely resembled those of the corresponding plants under drought stress. Few studies have investigated how root exudates are able to recover after drought (none to our knowledge investigating the effect on the metabolome), but the amount of carbon released during root exudation has some capacity for recovery in the two studies that have measured it under recovery after a previous drought period^32,35^.

## Conclusions

This study is the first to document the changes in the metabolome of root exudates subjected to varying drought levels and then subsequent recovery. We have demonstrated that even small changes in water availability can have a measurable and long-lasting impact on the composition of root exudates, and we have highlighted the compounds whose synthesis is activated at different stages of water stress.

Exudates of drought-stressed plants (except for those with the longest drought) tended to be dominated by secondary metabolites, although the precise composition varied depending on the intensity of the drought, and under the most extreme drought higher concentrations of fructose, arginine, and valine were seen. We did not find evidence for recovery of the root exudate metabolome after six weeks of re-watering, and we also demonstrated that the metabolite composition of root exudates during recovery after drought varied with the severity of the drought.

Overall, changes in root exudation composition, both during drought and recovery, are highly dependent on the severity of water stress. There may also be a threshold level of stress for plants beyond which they cannot recover, and that could be determined from the metabolome of the exudates. We propose that plant root exudate composition reflects the composition of the rest of the plant, with plants producing exudates using the most abundant metabolites available at the time, and therefore varies depending on environmental conditions. Exciting new developments in the collection and analysis of root exudates may lead to greater insight into the response of belowground plant tissues to abiotic stress and into the causes underlying species differences in drought tolerance.

## Materials and Methods

### Description of drought experiment

#### Plant and soil material

A greenhouse experiment was established in the experimental fields of the Autonomous University of Barcelona in May 2015. The experiment comprised 144 three-year-old *Q. ilex* saplings, approximately 80 cm in height and total biomass of 50 g (supplied by Forestal Catalana). The sapling roots were carefully washed in water prior to replanting to remove the previous soil so that the soil communities would be representative of the new soil. These plants were then re-potted in 3.5-L pots, with forty-eight in each of three soil treatments: control, drought-stressed and sterilised. All potting substrates contained 45% autoclaved peat, 45% of sand, and 10% of natural soil inoculum. The soil inoculum was collected from Prades Natural Park, which is the site of a long-term drought experiment (established in 1999) that reduces rainfall by approximately 30%. For details of the site see Peñuelas *et al*.^93^. The experiment was designed to include three different soil inocula representing three different soil histories. Firstly, ‘control’ soil inoculum was collected from the topsoil of control plots of the long-term drought experiment, to represent a natural holm oak forest soil under ambient precipitation. Secondly, ‘drought-stressed’ soil inoculum was taken from topsoil of the drought plots of the same long-term drought experiment. There was a third ‘sterilised’ soil inoculum, which used soil from the control plots of the long-term experiment that was later autoclaved to remove the majority of microorganisms in the soil. However, there was no significant difference in metabolomic profiles of our root exudate samples from plants grown in the different soil types (results of the PERMANOVA), so data was pooled into one group for the analyses presented in this paper. This resulted in there being 18 replicates at each drought level, with nine undergoing drought only and nine being kept for recovery. The plants were given adequate water (top-watering once a day to attain a soil moisture content of approximately 20–25%) for five weeks to allow them to adjust to the greenhouse environment. Mean air temperature during the experiment was 26.7 °C (monitored using ELUSB-2 data logger, Lascar Electronics, Wiltshire, UK). Soil temperature was monitored at a fine scale in five pots, across the different soil types (using a Decagon Em50 data logger with 5TM soil probes, Decagon Devices, Pullman, WA, USA), and the mean temperature was 27.0 °C.

#### Experimental design

**Figure 4:**
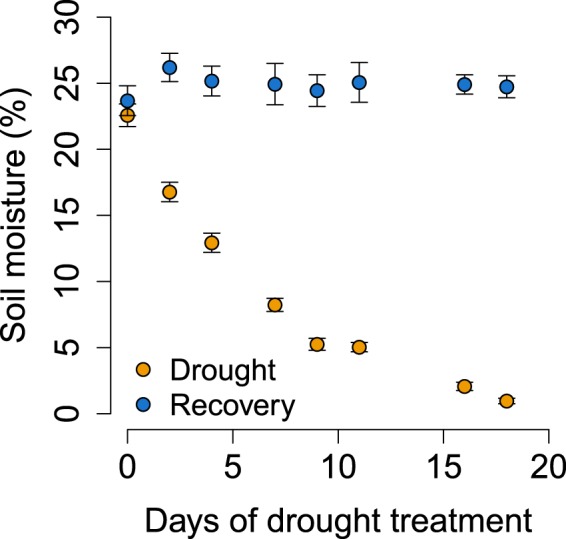
Changes in mean soil moisture (%) in response to (in yellow) the drought treatment (8 levels) and (in blue) the subsequent 6 week recovery period. Error bars are one SE.

The pots were subjected to drought by stopping the addition of water. Eight levels of drought were determined by the length of time without water: 0, 2, 4, 7, 9, 11, 16, and 18 days. Each drought level therefore had 18 pots, divided between the three soil treatments. These pots were arranged into six blocks. Root exudate samples were collected (method described below) from half of the pots at the end of each drought period and are hereafter referred to as “drought-stressed plants”. The remaining pots entered a six-week recovery phase with optimal watering and are hereafter referred to as “recovery” plants. Mean soil moisture (measured using ML3 Theta Probe connected to a HH2 Moisture Meter from Delta-T Devices, Cambridge, UK) at the start of the experiment was 22.6% and it decreased exponentially to 1.0% at the end of the most extreme drought treatment. Soil moisture recovered quickly to ca. 20% within one week of re-watering (top-watering as required to attain a soil moisture content of approximately 20–25%) and was 24.7% on average during the recovery phase (Fig. 4).

To measure the impact of the drought treatment and recovery on the aboveground part of the plants, chlorophyll fluorescence measurements were taken. For each level of drought, plants were measured 1–3 days before the collection of exudates during both the drought period and the recovery period. Three healthy and mature leaves at the top of each plant were measured at midday using a portable chlorophyll fluorometer PAM-2500 (H. Walz, Effeltrich, Germany). A saturating light pulse was applied to dark-adapted (at least 30 mins) leaves for the determination of minimum (*F*_o_) and maximum (*F*_m_) fluorescence. The maximum photochemical efficiency of photosystem II (PSII) was then calculated according to Genty *et al*.^94^ as *F*_v_/*F*_m_ = (*F*_m_ − *F*_o_)/*F*_m_.

#### Collection and preparation of exudates samples

Rhizodeposition was measured at the end of each drought period (for unwatered plants) and then at the end of each six-week recovery period (for re-watered plants) using a direct measuring technique adapted from that of Phillips *et al*. (2008). Briefly, a root was carefully excavated from the soil, cleaned, placed in moist sand, and then wrapped in aluminium foil. After one day of acclimation, the root was cleaned and placed in a cuvette containing small glass beads (to apply physical pressure to the root, as if it were in soil) and a carbon-free nutrient solution to prevent desiccation (0.5 mm NH_4_NO_3_, 0.1 mm KH_2_PO_4_, 0.2 mm K_2_SO_4_, 0.4 mm CaCl_2_, 0.15 mm MgSO_4_). The nutrient solution was replaced after two days with fresh solution (0.2 mm K_2_SO_4_, 0.4 mm CaCl_2_, 0.15 mm MgSO_4_), which was collected for analysis approximately 24 h later. Samples were filtered (0.2 µm pore size) and then stored at −20 °C until further analysis.

#### Extraction of metabolites for LC–MS analysis

We extracted the metabolites as described by t’Kindt *et al*. (2008) with minor modifications. The extraction solvent contained MeOH:H_2_O (80:20), which can recover a variety of metabolites such as polar and semipolar compounds, e.g. saccharides, amino acids, terpenoids, polysaccharides, flavonoids, and many other secondary metabolites. One millilitre of MeOH/H_2_O was added to Eppendorf tubes containing 150 mg of powder from each sample. The tubes were vortexed for 10 min, sonicated for 5 min at room temperature, and then centrifuged at 23 000 g for 5 min. After centrifugation, 0.7 mL of the supernatant from each tube was collected using crystal syringes, filtered through 0.22-µm pore microfilters, and transferred to a labelled set of high-performance liquid chromatography (HPLC) vials. The vials were stored at −80 °C until the LC–MS analysis. This procedure was repeated for two extractions of the same sample.

#### LC–MS analysis

The polar fraction of each sample was analysed twice on an Orbitrap LC-MS, once with the electrospray ionisation source operating in negative ionisation mode (−H) and once in positive ionisation mode (+H). Recordings from both the diode array detector and the high-resolution mass spectrometer were monitored and saved to verify system operation and evaluate later results.

LC–MS chromatograms were obtained with a Dionex Ultimate 3000 HPLC system (Thermo Fisher Scientific/Dionex RSLC, Dionex, Waltham, USA) coupled to an LTQ Orbitrap XL high-resolution mass spectrometer (Thermo Fisher Scientific, Waltham, USA) equipped with an HESI II (heated electrospray ionisation) source. Chromatography was performed on a reversed-phase C18 Hypersil gold column (150 × 2.1 mm, 3-µm particle size; Thermo Scientific, Waltham, USA) at 30 °C. The mobile phases consisted of acetonitrile (A) and water containing 0.1% acetic acid (B). Both mobile phases were filtered and degassed for 10 min in an ultrasonic bath prior to use. The elution gradient, at a flow rate of 0.3 mL min^−1^, began at 10% A (90% B) and was maintained for 5 min. The content of mobile phase A increased to 90% during the subsequent 15 minutes. This composition was then maintained for 5 min, after which the system was over a period of 5 min gradually equilibrated to the initial proportions (10% A and 90% B). The column was then washed and stabilised for 5 min before the next sample was injected. The injection volume of the samples was 5 µL. HESI was used for MS detection. The Orbitrap mass spectrometer was operated in Fourier Transform Mass Spectrometry mode, and full-scan spectra were acquired for mass range m/z 50–1000 in the positive mode and 65–1000 in the negative mode at high-mass resolution (60 000). The resolution and sensitivity of the Orbitrap were controlled by the injection of a mixed standard after the analysis of each batch (30 samples), and resolution was also checked with the aid of lock masses (phthalates). Blank samples were also analysed during the sequence. The assignment of the metabolites was based on the standards, and the metabolites were identified against our in-house library (>200 compounds) and manually annotated with the retention time (RT) and mass of the assigned metabolites in both positive and negative ionisation modes (Table 4).

**Table 4:** The 65 metabolites identified in roots exudates of *Quercus ilex*. Their abbreviations, relative retention times and mass to charge (m/z) ratios used for identification and quantification are also depicted. RT, retention time.

No.	Metabolites	Abbreviation	RT(min)	Quantifier ion		No.	Metabolites	Abbreviation	RT(min)	Quantifier ion	
				m/z	Type					m/z	Type
1	Asparagine	Asn	1.46	133.060	[M + H]^+^	34	Trans-Ferulic acid	Fer	10.57	217.0476	[M + H]^+^
2	Aspartic acid	Asp	1.47	148.060	[M + H]^+^	35	Syringic acid	Syr	4.9	199.0597	[M + H]^+^
3	Serine	Ser	1.47	106.049	[M + H]^+^	36	Oxaloglutarate	Oxa	1,46 and 1,77	282.08417	[M − H]^−^
4	Alanine	Ala	1.43	90.054	[M + H]^+^	37	Jasmonic acid (JA)	JA	14.81	211.13239	[M + H]^+^
5	Arginine	Arg	1.34	175.119	[M + H]^+^	38	Chlorogenic acid	Chl	3,11	355.0840	[M + H]^+^
6	Proline	Pro	1.49	116.070	[M + H]^+^	39	(−)-Abscisic acid	Abs	13.57	265.14334	[M + H]^+^
7	Valine	Val	1.53	118.086	[M + H]^+^	40	Choline	Cho	1.44	149.04608	[M − H]^−^
8	Isoleucine	Iso	1.70	132.101	[M + H]^+^	41	Sodium salicylate	Sali	11,09	305.0649	[M + H]^+^
9	Leucine	Leu	1.76	132.101	[M + H]^+^	42	Uracil	Ura	1.5	113.03413	[M + H]^+^
10	Glutamine	Gln	1.46	147.076	[M + H]^+^	43	Adenine	Ade	1,42 and 1,77	136.06143	[M + H]^+^
11	Methionin	Met	1.59	150.058	[M + H]^+^	44	Thymidine	Thy	1,48 and 1,81	243.09775	[M + H]^+^
12	Lysine	Lys	1.32	147.112	[M + H]^+^	45	Adenosine	Aden	1,49 and 1,75	268.10382	[M + H]^+^
13	Phenylalanine	Phe	1.91	166.086	[M + H]^+^	46	D-Ribose	Rib	1.43	149.04562	[M − H]^−^
14	Tyrosine	Tyr	1.54 a 1.77	182.081	[M + H]^+^	47	D-Lyxose	Lyx	1.44	149.04559	[M − H]^−^
15	Tryptophan	Trp	2.49	205.097	[M + H]^+^	48	D-Xylose	Xyl	1.77	191.01962	[M − H]^−^
16	Succinic Acid	SucA	1,74, 1,78	117.01942	[M − H]^−^	49	D-Fructose	Fru	1.42	149.04564	[M − H]^−^
17	Malic acid	Mal	10.57	193.0513	[M − H]^−^	50	5-Methylthio-D-ribose	RibO	1.43	149.04562	[M − H]^−^
18	Citrate	Cit	1.75	193.03372	[M + H]^+^	51	D-Sorbose	Sor	1.65	87.00892	[M − H]^−^
19	Lactic acid	Lac	11.29	163.0402	[M − H]^−^	52	D-Glucose	Gluc	1.44	179.05602	[M − H]^−^
20	Quinic acid	Quin	1.51	193.07074	[M + H]^+^	53	D-Galactose	Gae	1.65	87.00892	[M − H]^−^
21	Pyruvate	Pyr	7.71	87.054	[M − H]^−^	54	D-(+)-Trehalose dihydrate	Trha	3.11	353.0884	[M − H]^−^
22	p-Coumaric acid	CouA	11.29	187.0366	[M + H]^+^	55	Resveratrol	Res	13.09	229.0857	[M + H]^+^
23	Jasmone	Jas	16.63	155.1424	[M + H]^+^	56	Acacetin	Aca	16.87	285.07547	[M + H]^+^
24	Vanillic acid	Van	4.65	169.0429	[M + H]^+^	57	Kaempferol	Kae	14.82	287.0552	[M + H]^+^
25	Gallic acid	Gal	1,55 a 1,83	171.0224	[M + H]^+^	58	Epicatechin	Epi	4,93 a 5,20	291.0862	[M + H]^+^
26	Maleic acid	MaA	8.96	117.072	[M + H]^+^	59	Quercetin	Que	13.72	303.0498	[M + H]^+^
27	Fumarate	Fum	9.63	115.056	[M + H]^+^	60	Epigallocatechin	Epg	1,54 a 2,64	307.0812	[M + H]^+^
28	Succinic acid	Suc	1.47	106.049	[M + H]^+^	61	Myricetin	Myr	12.47	319.0446	[M + H]^+^
29	alpha-Ketoglutaric acid	Ket	1,42 and 1,64	168.0665	[M − H]^−^	62	Aucubine	Auc	1.45	151.06148	[M − H]^−^
30	Cinnamic acid	Cin	14.28	149.059	[M + H]^+^	63	Isovitexin	Isx	11.1	433.1125	[M + H]^+^
31	trans-Caffeic acid	Caf	4.82	181.0551	[M + H]^+^	64	Homoorientin	Hom	9.45	449.1078	[M + H]^+^
32	Citric acid (anhydrous)	CitA	1.75	193.03372	[M + H]^+^	65	Lucenin	Luc	1.76	132.101	[M + H]^+^
33	Quinic acid	QA	1.51	193.07074	[M + H]^+^						

#### Processing of LC–MS

The raw LC–MS data files were processed using an open source software MZMINE 2.10 (Pluskal *et al*., 2010) (see Table S1 for details). The chromatograms were baseline corrected, deconvoluted, aligned, and filtered, and the numerical database was then exported in “csv” format. Metabolites were assigned by comparison with the analyses of the standards (RT and mass) (see Table S1 for details). Assigned variables corresponding to the same molecular compounds were summed. The LC–MS data for the statistical analyses corresponded to the absolute peak area at each RT. The area of a peak is directly proportional to the concentration (i.e. µg mL^−1^) of its corresponding (assigned) metabolite in the sample, so a change in the area of a peak indicates a change in the concentration of its assigned metabolite. Most metabolites were related to important metabolic pathways. The increases and decreases in concentrations of these metabolites showed up and down regulation in these metabolic pathways.

#### Statistical analyses

The LC–MS data were analysed by univariate and multivariate statistical analyses. We conducted permutational multivariate analyses of variance (PERMANOVAs) (Anderson *et al*., 2008) using Euclidean distances, with drought/recovery and drought intensity (eight levels of drought) as fixed factors and individuals as random factors. Partial least squares discriminant analyses (PLSDAs) were performed to detect patterns of sample ordination in the metabolomic variables. The PLSDAs were initially constructed from the LC–MS analysis of exudates to enable the identification of clusters, groups, and outliers (Sandasi *et al*., 2011) (Fig. 2). The PC scores of the cases were subjected to one-way ANOVAs to determine the statistical differences among groups with different levels of the categorical independent variables (drought and recovery). The PERMANOVAs and PLSDAs used the *mixOmics* package of R (R Development Core Team 2015). The Kolmogorov-Smirnov (KS) test was performed on each variable to test for normality. All assigned and identified metabolites were normally distributed, and any unidentified metabolomic variable that was not normally distributed was removed from the data set. Statistica v8.0 was used for the ANOVAs, post hoc tests, and KS tests. Chlorophyll fluorescence data were analysed using repeated-measures analyses of variance (ANOVAs), with differences considered statistically significant at *P* < 0.05 (R Development Core Team 2015).

## Electronic supplementary material

Supplementary information

## References

[1] Bais HP, Weir TL, Perry LG, Gilroy S, Vivanco JM (2006). The Role of Root Exudates in Rhizosphere Interactions With Plants and Other Organisms. Annu. Rev. Plant Biol..

[2] Philippot L, Raaijmakers JM, Lemanceau P, van der Putten WH (2013). Going back to the roots: the microbial ecology of the rhizosphere. Nat Rev Micro.

[3] Zhu B (2014). Rhizosphere priming effects on soil carbon and nitrogen mineralization. Soil Biol. Biochem..

[4] Keiluweit M (2015). Mineral protection of soil carbon counteracted by root exudates. Nat. Clim. Chang..

[5] Rousk K, Michelsen A, Rousk J (2016). Microbial control of soil organic matter mineralization responses to labile carbon in subarctic climate change treatments. Glob. Chang. Biol..

[6] Badri DV, Vivanco JM (2009). Regulation and function of root exudates. Plant. Cell Environ..

[7] Baetz U, Martinoia E (2014). Root exudates: The hidden part of plant defense. Trends Plant Sci..

[8] Dakora FD, Phillips DA (2002). Root exudates as mediators of mineral acquisition in low-nutrient environments. Plant Soil.

[9] Faure D, Vereecke D, Leveau JHJ (2009). Molecular communication in the rhizosphere. Plant Soil.

[10] Jones DL, Hodge A, Kuzyakov Y (2004). Plant and mycorrhizal regulation of rhizodeposition. New Phytol..

[11] Pinton, R., Varanini, Z. & Nannipieri, P. *The rhizosphere: biochemistry and organic substances at the soil-plant interface*. (CRC press, 2007).

[12] Jones DL, Nguyen C, Finlay RD (2009). Carbon flow in the rhizosphere: carbon trading at the soil–root interface. Plant Soil.

[13] Kuzyakov Y, Domanski G (2000). Carbon input by plants into the soil. Review. J. Plant Nutr. Soil Sci..

[14] Ma JF, Ryan PR, Delhaize E (2001). Aluminium tolerance in plants and the complexing role of organic acids. Trends Plant Sci..

[15] Hirsch AM (2003). Molecular signals and receptors: controlling rhizosphere interactions between plants and other organisms. Ecology.

[16] Rudrappa T, Czymmek KJ, Paré PW, Bais HP (2008). Root-secreted malic acid recruits beneficial soil bacteria. Plant Physiol..

[17] Gargallo-Garriga A (2016). Shifts in plant foliar and floral metabolomes in response to the suppression of the associated microbiota. BMC Plant Biol..

[18] Biedrzycki ML, Jilany TA, Dudley SA, Bais HP (2010). Root exudates mediate kin recognition in plants. Commun. Integr. Biol..

[19] Aulakh MS, Wassmann R, Bueno C, Kreuzwieser J, Rennenberg H (2001). Characterization of root exudates at different growth stages of ten rice (Oryza sativa L.) cultivars. Plant Biol..

[20] Chaparro JM (2013). Root exudation of phytochemicals in Arabidopsis follows specific patterns that are developmentally programmed and correlate with soil microbial functions. PLoS One.

[21] Neumann G, Römheld V (1999). Root excretion of carboxylic acids and protons in phosphorus-deficient plants. Plant Soil.

[22] Johnson JF, Allan DL, Vance CP (1994). Phosphorus stress-induced proteoid roots show altered metabolism in Lupinus albus. Plant Physiol..

[23] Lipton DS, Blanchar RW, Blevins DG (1987). Citrate, malate, and succinate concentration in exudates from P-sufficient and P-stressed Medicago sativa L. seedlings. Plant Physiol..

[24] Hoffland E, Van Den Boogaard R, Nelemans J, Findenegg G (1992). Biosynthesis and root exudation of citric and malic acids in phosphate-starved rape plants. New Phytol..

[25] Li YP, Ye W, Wang M, Yan XD (2009). Climate change and drought: a risk assessment of crop-yield impacts. Clim. Res..

[26] Dai A (2011). Drought under global warming: a review. Wiley Interdiscip. Rev. Clim. Chang..

[27] Ryan MG (2011). Tree responses to drought. Tree Physiol..

[28] Harfouche A, Meilan R, Altman A (2014). Molecular and physiological responses to abiotic stress in forest trees and their relevance to tree improvement. Tree Physiol..

[29] Brunner I, Herzog C, Dawes MA, Arend M, Sperisen C (2015). How tree roots respond to drought. Front. Plant Sci..

[30] Cuneo IF, Knipfer T, Brodersen CR, McElrone AJ (2016). Mechanical Failure of Fine Root Cortical Cells Initiates Plant Hydraulic Decline during Drought. Plant Physiol..

[31] Preece C, Peñuelas J (2016). Rhizodeposition under drought and consequences for soil communities and ecosystem resilience. Plant Soil.

[32] Preece, C., Farré-Armengol, G., Llusià, J. & Peñuelas, J. Thirsty tree roots exude more carbon. *Tree Physiol*. **38**(5), 690–695 10.1093/treephys/tpx163 (2018). 10.1093/treephys/tpx163 29304257

[33] Czarnes S, Hallett PD, Bengough AG, Young IM (2000). Root- and microbial-derived mucilages affect soil structure and water transport. Eur. J. Soil Sci..

[34] Ahmed MA, Kroener E, Holz M, Zarebanadkouki M, Carminati A (2014). Mucilage exudation facilitates root water uptake in dry soils. Funct. Plant Biol..

[35] Fuchslueger L, Bahn M, Fritz K, Hasibeder R, Richter A (2014). Experimental drought reduces the transfer of recently fixed plant carbon to soil microbes and alters the bacterial community composition in a mountain meadow. New Phytol..

[36] Dyer CL, Kopittke PM, Sheldon AR, Menzies NW (2008). Influence of soil moisture content on soil solution composition. Soil Sci. Soc. Am. J..

[37] Gargallo-Garriga A (2014). Opposite metabolic responses of shoots and roots to drought. Sci. Rep..

[38] Gargallo-Garriga A (2015). Warming differentially influences the effects of drought on stoichiometry and metabolomics in shoots and roots. New Phytol..

[39] Canarini A, Dijkstra FA (2015). Dry-rewetting cycles regulate wheat carbon rhizodeposition, stabilization and nitrogen cycling. Soil Biol. Biochem..

[40] Song FB, Han XY, Zhu XC, Herbert SJ (2012). Response to water stress of soil enzymes and root exudates from drought and non-drought tolerant corn hybrids at different growth stages. Can. J. Soil Sci..

[41] Henry A, Doucette W, Norton J, Bugbee B (2007). Changes in crested wheatgrass root exudation caused by flood, drought, and nutrient stress. J. Environ. Qual..

[42] van Dam NM, Bouwmeester HJ (2016). Metabolomics in the rhizosphere: tapping into belowground chemical communication. Trends Plant Sci..

[43] Valentinuzzi F (2015). Phosphorus and iron defciencies induce a metabolic reprogramming and affect the exudation traits of the woody plant Fragaria x ananassa. J. Exp. Bot..

[44] Zubair HM, Pratley JE, Sandral GA, Humphries A (2017). Allelopathic interference of alfalfa (Medicago sativa L.) genotypes to annual ryegrass (Lolium rigidum). J. Plant Res..

[45] Canarini A, Merchant A, Dijkstra FA (2016). Drought effects on Helianthus annuus and Glycine max metabolites: from phloem to root exudates. Rhizosphere.

[46] Peñuelas J, Sardans J (2009). Ecological metabolomics. Chem. Ecol..

[47] Sardans J, Peñuelas J, Rivas-Ubach A (2011). Ecological metabolomics: overview of current developments and future challenges. Chemoecology.

[48] Fiehn O (2002). Metabolomics–the link between genotypes and phenotypes. Plant Mol. Biol..

[49] Gargallo-Garriga A (2017). Long-term fertilization determines different metabolomic profiles and responses in saplings of three rainforest tree species with different adult canopy position. PLoS One.

[50] Gargallo-Garriga A (2017). Impact of soil warming on the plant metabolome of icelandic grasslands. Metabolites.

[51] Lloret F, Peñuelas J, Ogaya R (2004). Establishment of co-existing Mediterranean tree species under a varying soil moisture regime. J. Veg. Sci..

[52] Sánchez-Humanes, B. & Espelta, J. M. Increased drought reduces acorn production in Quercus ilex coppices: thinning mitigates this effect but only in the short term. *Forestry*, 10.1093/forestry/cpq045 (2011).

[53] Rivas-Ubach A (2014). Drought enhances folivory by shifting foliar metabolomes in Quercus ilex trees. New Phytol..

[54] Angelica MD, Fong Y (2008). NIH Public Access. October.

[55] Curiel-Yuste J (2011). Drought-resistant fungi control soil organic matter decomposition and its response to temperature. Glob. Chang. Biol..

[56] Dam NMV, Bouwmeester HJ (2016). Metabolomics in the rhizosphere: tapping into belowground chemical communication. Trends Plant Sci..

[57] Bilgin DD (2010). Biotic stress globally downregulates photosynthesis genes. Plant, Cell Environ..

[58] Wilkinson S, Davies WJ (2002). ABA-based chemical signalling: The co-ordination of responses to stress in plants. Plant, Cell Environ..

[59] Wilkinson S, Davies WJ (2010). Drought, ozone, ABA and ethylene: new insights from cell to plant to community. Plant, Cell Environ..

[60] Davies WJ, Zhang JH (1991). Root signals and the regulation of growth and development of plants in drying soil. Annu. Rev. Plant Physiol. Plant Mol. Biol..

[61] McAdam SAM, Manzi M, Ross JJ, Brodribb TJ, Gómez-Cadenas A (2016). Uprooting an abscisic acid paradigm: Shoots are the primary source. Plant Signal. Behav..

[62] Manzi M, Pitarch-Bielsa M, Arbona V, Gomez-Cadenas A (2017). Leaf dehydration is needed to induce abscisic acid accumulation in roots of citrus plants. Environ. Exp. Bot..

[63] de Ollas C, Arbona V, Gómez-Cadenas A (2015). Jasmonoyl isoleucine accumulation is needed for abscisic acid build-up in roots of Arabidopsis under water stress conditions. Plant, Cell Environ..

[64] Sardans J (2013). Metabolic responses of Quercus ilex seedlings to wounding analysed with nuclear magnetic resonance profiling. Plant Biol..

[65] Li Z, Yu J, Peng Y, Huang B (2017). Metabolic pathways regulated by abscisic acid, salicylic acid and γ-aminobutyric acid in association with improved drought tolerance in creeping bentgrass (Agrostis stolonifera). Physiol. Plant..

[66] Li X, Dong J, Chu W, Chen Y (2018). The relationship between root exudation properties and root morphological traits of cucumber grown under different nitrogen supplies and atmospheric CO 2 concentrations. Plant Soil.

[67] Lesuffleur F, Cliquet JB (2010). Characterisation of root amino acid exudation in white clover (Trifolium repens L.). Plant Soil.

[68] Good AG, Zaplachinski ST (1994). The effects of drought stress on free amino acid accumulation and protein synthesis in Brassica napus. Physiol. Plant..

[69] De Diego N (2013). Solute accumulation and elastic modulus changes in six radiata pine breeds exposed to drought. Tree Physiol..

[70] Wu GQ, Feng RJ, Shui QZ (2016). Effect of osmotic stress on growth and osmolytes accumulation in sugar beet (Beta vulgaris L.) plants. Plant Soil Environ..

[71] Hare PD, Cress WA, Van Staden J (1998). Dissecting the roles of osmolyte accumulation during stress. Plant. Cell Environ..

[72] Serraj R, Sinclair TR (2002). Osmolyte accumulation: can it really help increase crop yield under drought conditions?. Plant. Cell Environ..

[73] Blum A (2017). Osmotic adjustment is a prime drought stress adaptive engine in support of plant production. Plant Cell Environ..

[74] Gregorová Z (2015). Drought-induced responses of physiology, metabolites, and PR proteins in Triticum aestivum. J. Agric. Food Chem..

[75] Chmielewska K (2016). Analysis of drought-induced proteomic and metabolomic changes in barley (Hordeum vulgare L.) leaves and roots unravels some aspects of biochemical mechanisms involved in drought tolerance. Front. Plant Sci..

[76] Bhaskara GB, Yang T-H, Verslues PE (2015). Dynamic proline metabolism: importance and regulation in water limited environments. Front. Plant Sci..

[77] Macková H (2013). Enhanced drought and heat stress tolerance of tobacco plants with ectopically enhanced cytokinin oxidase/dehydrogenase gene expression. J. Exp. Bot..

[78] Xu, Y., Burgess, P. & Huang, B. Transcriptional regulation of hormone-synthesis and signaling pathways by overexpressing cytokinin-synthesis contributes to improved drought tolerance in creeping bentgrass. *Physiol. Plant*. **161**(2), 235–256. 10.1111/ppl.12588 (2017). 10.1111/ppl.12588 28543596

[79] Kang Y (2011). System responses to long-term drought and re-watering of two contrasting alfalfa varieties. Plant J..

[80] Agati G, Azzarello E, Pollastri S, Tattini M (2012). Flavonoids as antioxidants in plants: Location and functional significance. Plant Sci..

[81] Peñuelas J, Estiarte M, Kimball B (1999). Flavonoid responses in wheat grown at elevated CO2: green versus senescent leaves. Photosynthetica.

[82] Nakabayashi R (2014). Enhancement of oxidative and drought tolerance in Arabidopsis by overaccumulation of antioxidant flavonoids. Plant J..

[83] Kape R (1991). Chemotaxis and nod Gene Activity of Bradyrhizobium japonicum in Response to Hydroxycinnamic Acids and Isoflavonoids. Appl. Environ. Microbiol..

[84] Stafford HA (1997). Roles of Flavonoids in Symbiotic and Defense Functions in Legume Roots. Bot. Rev..

[85] Aoki T, Akashi T, Ayabe S (2000). Flavonoids of Leguminous Plants: Structure, Biological Activity, and Biosynthesis. J. Plant Res..

[86] Forkmann G, Martens S (2001). Metabolic engineering and applications of flavonoids. Curr. Opin. Biotechnol..

[87] Watson BS (2015). Integrated Metabolomics and Transcriptomics Reveal Enhanced Specialized Metabolism in *Medicago truncatula* Root Border Cells. Plant Physiol..

[88] Zhang, J., Chen, G., Zhao, P., Zhou, Q. & Zhao, X. The abundance of certain metabolites responds to drought stress in the highly drought tolerant plant Caragana korshinskii. *Acta Physiol. Plant*. **39** (2017).

[89] Ta TC, Joy KW, Ireland R (1985). Role of asparagine in the photochemistry nitrogen metaboliem of pea leaves. Plant Physiol..

[90] Zhang C, Preece C, Filella I, Farré-Armengol G, Peñuelas J (2017). Assessment of the response of photosynthetic activity of Mediterranean evergreen oaks to enhanced drought stress and recovery by using PRI and R690/R630. Forests.

[91] Souza RP, Machado EC, Silva JAB, Lagôa AMMA, Silveira JAG (2004). Photosynthetic gas exchange, chlorophyll fluorescence and some associated metabolic changes in cowpea (Vigna unguiculata) during water stress and recovery. Environ. Exp. Bot..

[92] Miyashita K, Tanakamaru S, Maitani T, Kimura K (2005). Recovery responses of photosynthesis, transpiration, and stomatal conductance in kidney bean following drought stress. Environ. Exp. Bot..

[93] Peñuelas, J. *et al*. Assessment of the impacts of climate change on Mediterranean terrestrial ecosystems based on data from field experiments and long-term monitored field gradients in Catalonia. *Environmental and Experimental Botany* **152**, 49–59 10.1016/j.envexpbot.2017.05.012 (2018).

[94] Genty B, Jean-Marie B, Neil R. B (1989). The relationship between the quantum yield of photosynthetic electron transport and quenching of chlorophyll fluorescence. Biochim. Biophys. Acta (BBA)-General Subj..

