# Supplementary material for: Root exudate metabolomes change under drought and show limited capacity for recovery
Source: Sci Rep. 2018 Aug 23;8:12696. doi: 10.1038/s41598-018-30150-0 (PMC6107494; doi:10.1038/s41598-018-30150-0)
Supplement: Supplementary file 1 — Supplementary information [file 41598_2018_30150_MOESM1_ESM.docx]

**Root exudate metabolomes change under drought and show limited capacity for recovery**

**Albert Gargallo-Garriga*^1,2,3^, Catherine Preece*^1,2^, Jordi Sardans^1,2^, Michal Oravec^3^, Otmar Urban^3^, Josep Peñuelas^1,2^.**

# Supplementary information

**Table S1.** Processing parameters of the LC-MS chromatograms using MZmine 2.10 ^1^. The chromatograms correspond to the total ion current (TIC).

|  | (+H) Chromatograms |  | (-H) Chromatograms |
| --- | --- | --- | --- |
| **Baseline correction** |  |  |  |
| Chromatogram type | TIC |  | TIC |
| MS level | 1 |  | 1 |
| Smoothing | 10E6 |  | 10E6 |
| Asymmetry | 0.001 |  | 0.001 |
| **Mass detection** (Exact mass) |  |  |  |
| Noise level | 4.5 × 10^5^ |  | 4.5 × 10^5^ |
| **Chromatogram builder** |  |  |  |
| Min time span | 0.05 |  | 0.05 |
| Min height | 25 000 |  | 25 000 |
| m/z tolerance | 0.002 |  | 0.002 |
| **Smoothing** |  |  |  |
| Filter width | 5 |  | 5 |
| **Chromatogram deconvolution (Local minimum search)** |  |  |  |
| Chromatographic threshold | 70% |  | 70% |
| Search minimum in RT range (min) | 0.1 |  | 0.1 |
| Minimum relative height | 7.0% |  | 7.0% |
| Minimum absolute height | 30 000 |  | 30 000 |
| Min ratio of peak top/edge | 2 |  | 2 |
| Peak duration range | 0.0–2.0 |  | 0.0–2.0 |
| **Chromatogram alignment (join alignment)** |  |  |  |
| m/z tolerance | 0.001 |  | 0.001 |
| Weight for m/z | 80 |  | 80 |
| RT tolerance | 0.3 |  | 0.3 |
| Weight for RT | 20 |  | 20 |
| **Gap filling (Peak Finder)** |  |  |  |
| Intensity tolerance | 20% |  | 20% |
| m/z tolerance | 0.001 |  | 0.001 |
| Retention time tolerance | 0.1 |  | 0.1 |
| RT correction | marked |  | marked |
| **Filtering** |  |  |  |
| Minimum peaks in a row | 25 |  | 25 |
|  |  |  |  |
| **Ions excluded from database** | <75  Between 0.0 and 1 min  Between 28.5 and 30 min |  | <85  Between 0.0 and 1.1 min  Between 27.0 and 30 min |

Fig.1S. Response of individual metabolites in representative metabolic pathways to drought treatments measured as the fold difference between individual treatments and that in the recovery treatment. Red colour indicates activated (with increased concentrations) metabolites and blue colour indicates deactivated (with decreased concentrations) metabolites.

E-) Metabolites of different metabolic pathways that shifted their concentrations under the control and high drought (16 days with no water.


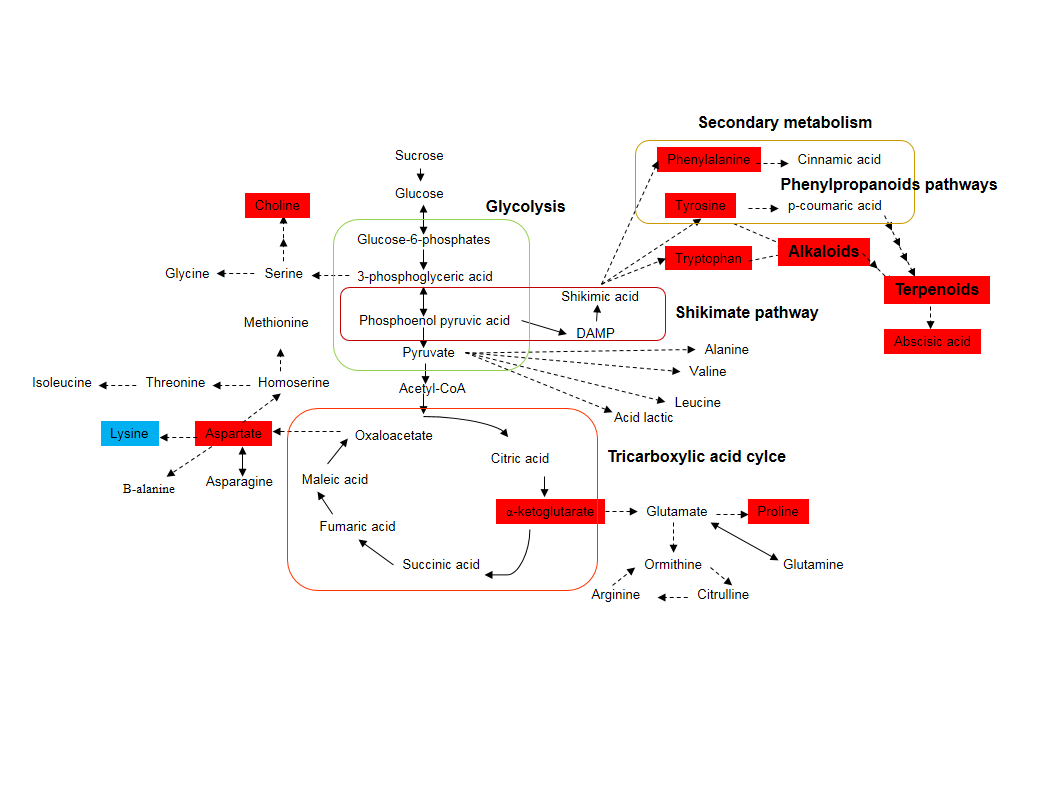


F-) Metabolites of different metabolic pathways that shifted their concentrations under the high drought (16 days with no water) and high recovery (16 days with water) in *Quercus Ilex*.


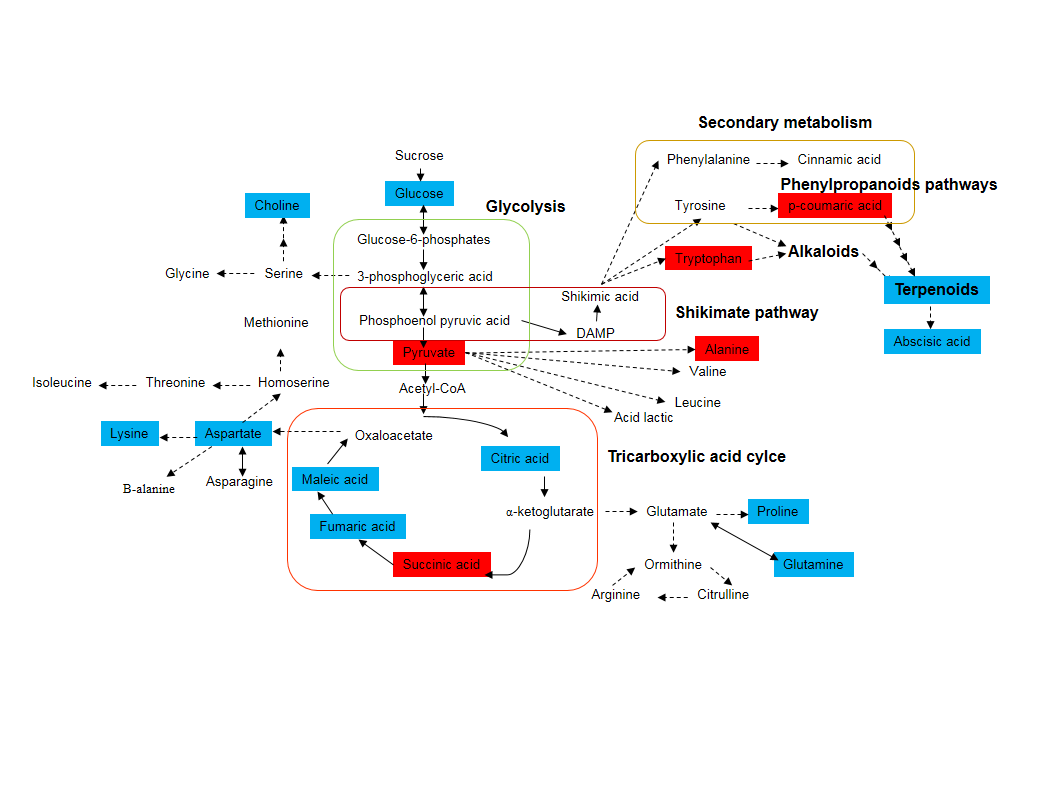


1. Pluskal, T., Castillo, S., Villar-Briones, A. & Oresic, M. MZmine 2: modular framework for processing, visualizing, and analyzing mass spectrometry-based molecular profile data. *BMC Bioinformatics* **11,** 395 (2010).
